# Characterization of fibroblast-like synoviocytes from the synovial fluid of patients affected by juvenile idiopathic arthritis

**DOI:** 10.1093/rheumatology/keaf266

**Published:** 2025-05-23

**Authors:** Simone Pelassa, Federica Raggi, Ignazia Prigione, Francesca Ridella, Angelo Ravelli, Marco Gattorno, Alessandro Consolaro, Maria Carla Bosco

**Affiliations:** UOC Rheumatology and Autoinflammatory Diseases, Department of Pediatric Sciences, IRCCS Istituto Giannina Gaslini, Genova, Italy; UOC Rheumatology and Autoinflammatory Diseases, Department of Pediatric Sciences, IRCCS Istituto Giannina Gaslini, Genova, Italy; UOC Rheumatology and Autoinflammatory Diseases, Department of Pediatric Sciences, IRCCS Istituto Giannina Gaslini, Genova, Italy; Department of Neurosciences, Rehabilitation, Ophthalmology, Genetics and Maternal Infantile Sciences (DiNOGMI), University of Genova, Genova, Italy; Scientific Direction, IRCCS Istituto Giannina Gaslini, Genova, Italy; UOC Rheumatology and Autoinflammatory Diseases, Department of Pediatric Sciences, IRCCS Istituto Giannina Gaslini, Genova, Italy; UOC Rheumatology and Autoinflammatory Diseases, Department of Pediatric Sciences, IRCCS Istituto Giannina Gaslini, Genova, Italy; Department of Neurosciences, Rehabilitation, Ophthalmology, Genetics and Maternal Infantile Sciences (DiNOGMI), University of Genova, Genova, Italy; UOC Rheumatology and Autoinflammatory Diseases, Department of Pediatric Sciences, IRCCS Istituto Giannina Gaslini, Genova, Italy

**Keywords:** paediatric rheumatology, juvenile idiopathic arthritis, fibroblast-like synoviocytes, auto-inflammation

## Abstract

**Objectives:**

The objectives of this study were to characterize some phenotypic and functional aspects of fibroblast-like synoviocytes isolated from the Synovial Fluid (SF-FLSs) of patients affected by Juvenile Idiopathic Arthritis (JIA) with active disease and to compare SF-FLS characteristics with those reported in the literature for FLSs of the SM (Synovial Membrane) in adult rheumatic patients.

**Methods:**

FLSs were isolated from the SF of JIA patients with active disease by therapeutic arthrocentesis. SF-FLS surface marker expression was assessed by cytofluorimetry; proinflammatory cytokine and *MMP* gene expression was investigated by quantitative RT-PCR (qRT-PCR); and chondrogenic properties were evaluated by staining with Alcian-Blue.

**Results:**

SF-FLSs display a CD45^−^,CD34^−^,CD90^+^,PDPN^+^ phenotype and exhibit a significant increase in the expression of genes coding for pro-inflammatory cytokines, but not MMPs, when treated with TNF-α, a cytokine present in the joint environment. In addition, SF-FLSs express the chondrogenic genes BMP4 and Aggrecan (AGG) and show the ability to differentiate towards a chondrocyte-like phenotype when cultured in TGF-β–enriched medium.

**Conclusion:**

FLSs from the SF of JIA patients display a phenotype that has pro-inflammatory, rather than tissue-disruptive, activity; this is similar to what is observed in cells from the sublining region of the SM in adult arthritis patients. In addition, they show chondrogenic ability, indicating the potential for SF-FLSs as an *in vitro* model for chondrocytes.

## Graphical Abstract

**Figure:**
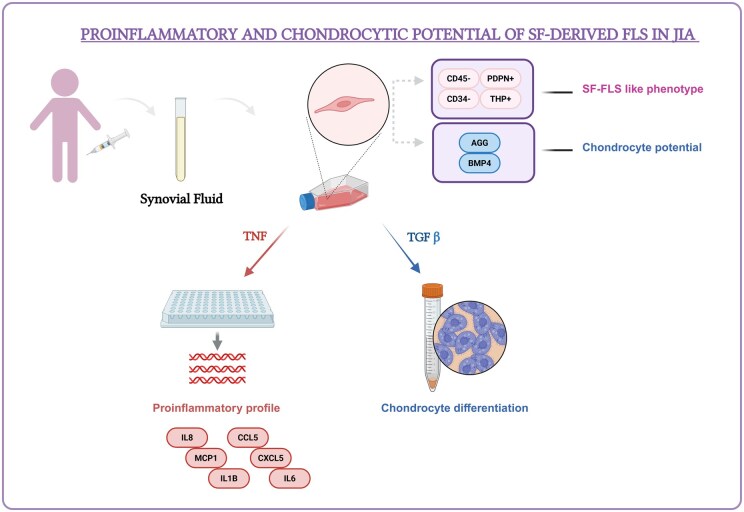

Rheumatology key messages

- FLSs from JIA patients show a phenotype similar to that observed in cells of the sublining region of the synovial membrane.
- FLSs from JIA patients display inflammatory characteristics rather than tissue-destructive properties.
- FLSs from JIA patients retain chondrogenic properties and could be potentially helpful as an in vitro model for chondrocytes.

## Introduction

JIA is an umbrella term used to indicate a clinically heterogeneous group of chronic paediatric rheumatic disorders characterized by unknown aetiology, persistent joint inflammation, onset before 16 years of age, and persistence for >6 weeks, which represent an important cause of child disability [1–3]. According to the classification of ILAR [4], JIA patients are grouped into seven different categories based on clinical manifestations during the first 6 months of disease: systemic arthritis, OA, RF-positive polyarthritis, RF-negative polyarthritis, enthesitis-related arthritis, PsA, and undifferentiated arthritis [5]. Both resident and infiltrating cells in the synovium play a central role in the orchestration of inflammatory and proliferative responses in the joints of JIA patients [6, 7]. As a consequence of inflammation and uncontrolled cell proliferation, the physiological structure of the SM is altered, resulting in pronounced hyperplasia of the lining layer (LL) and persistent infiltration of the sublining layer (SL) by mononuclear leukocytes, with consequent joint destruction and functional impairment [8]. Fibroblast-like synoviocytes (FLSs) represent one of the main stromal cell populations within the joint SM and are critically involved in JIA pathogenesis [9–11].

To date, several groups have studied FLSs in the SM of patients affected by different types of adult arthritides and in murine models of rheumatic diseases [12–14]. According to the literature, FLSs in the SM can be separated into two main groups: those belonging to the SL (SL-FLS) and those belonging to the LL (LL-FLS) regions, which display different phenotypes, thus representing distinct subtypes [12]. Specifically, it was reported that SL-FLSs display a THY^+^PDPN^+^ phenotype, whereas LL-FLSs are THY^−^PDPN^+^ in both patients affected by RA and RA murine models [12, 13]. Moreover, SL- and LL-FLSs were shown to play different roles in disease progression: the former contribute to the orchestration of inflammatory responses within the joint through the release of cytokines/chemokines, while the latter are mostly involved in cartilage degradation through the release of MMPs [11, 12]. To date only a few studies have investigated the composition of the SM in patients affected by JIA, characterizing infiltrated immune cells but not the stromal components [8].

FLS-like cells have also been detected in the SF (SF-FLSs) of adult arthritic patients [15–17] and reported by Koster *et al.* to present the THY^+^PDPN^+^ phenotype and pro-inflammatory properties [16], although their origin is still under debate [18]. In addition, FLS-like cells isolated from the SF of adult rheumatic patients have been defined as mesenchymal stem cells (MSCs) and reported to retain totipotent properties, differentiating into chondrocytes when stimulated under specific conditions [19]. Interestingly, Orange and colleagues demonstrated the presence of PRe-Inflammatory MEsenchymal PDPN^+^ (PRIME) cells in the SF of RA patients during flare and in the Peripheral Blood (PB) 1 week before flare occurrence, suggesting that these cells extravasate from blood vessels into the joint, contributing to the inflammatory responses [20]. The issue of FLSs in JIA has been addressed only in part by Brescia and colleagues, who suggested their potential chondrogenic and proinflammatory properties [9, 10, 21].

The aim of this work was to provide a phenotypic and gene expression characterization of SF-FLSs isolated from JIA patients with active disease, in order to assess their similarities and differences with the LL-FLS or SL-FLS subpopulations detected in the SM of adult patients and, thus, to obtain novel insights into JIA pathophysiology. The ability of SF-FLSs to differentiate into chondrocytes was also investigated.

## Methods

### Study population

Seven patients fulfilling the 2001 ILAR classification criteria for various JIA subtypes and undergoing therapeutic arthrocentesis at the Unit of Rheumatology and Autoinflammatory Diseases, Department of Pediatric Sciences, IRCCS Istituto Giannina Gaslini, Genova, were enrolled in the study between 2018 and 2023. All patients had clinically active disease with joint effusion, swelling, pain, and stiffness at the time of sampling. The number of active joints was determined by standard clinical evaluation followed by US or MRI evaluation. Arthrocentesis was performed under local anaesthesia or, in case of younger patients or multiple joints, under general sedation. Five children not affected by rheumatic conditions and undergoing minor surgical procedures at the Gaslini Institute were enrolled as a control group. The main demographic and clinical characteristics of the study cohort at the time of sampling are reported in Table 1 and Supplementary Table S1.

**Table 1.** Demographic and clinical data for JIA patients and control children

	Patients (*n* = 7)	Control children (*n* = 5)
**Female** ^a^	5 (71%)	3 (60%)
**Age at sampling (years)** ^b^	15.31 (9.34–17.99)	12 (10–15)
**No. of active joints (at enrolment)** ^b^	2 (1–5)	
**Diagnosis** ^c^	OA (28.5%), Polyarthritis RF^−^ (57.3%), systemic JIA (14.2%)	
**Therapy** ^d^	None (28.6%), DMARDs (28.6%), Biologic (42.8%)	

a Results are expressed as number of patients (percentage in parentheses).

b Results are expressed as mean (range in parentheses).

c Results are expressed as JIA subtypes (percentage in parentheses).

d Drug therapies administered before sampling.

### SF sample collection, cell isolation and culture

SF aspirates were obtained by knee arthrocentesis and collected into tubes containing EDTA, as described by Raggi *et al.* in 2022 and 2023 [22, 23]. FLSs were isolated from the SF of patients, as detailed by Stebulis *et al.* [15]. Briefly, SF from patients undergoing arthrocentesis was centrifuged at 300 *g* for 10 min at room temperature (RT) within 2 h of collection, and the cell pellet was resuspended in DMEM (Euroclone, Milan, Italy) with added 10% Fetal Bovine Serum (FBS, Gibco, Waltham, USA), 1% Pen/Strep, and 1% Glutammine (Euroclone, Milan, Italy) (complete medium). Cells were cultured at 37°C and trypsinized when 80% confluence was reached. Primary cultures were passaged three times to ensure that FLSs were the predominant cell types. For stimulation experiments, cells were seeded at 3 × 10^4^/ml in 12-well plates and treated with TNF-α (10 ng/ml, Peprotech, Waltham, USA) for 24 h.

Skin fibroblasts (sFBs) from biopsies of children undergoing minor surgical procedures were used as controls (CTR).

### FACS characterization

SF-FLSs were resuspended in PBS supplemented with 0.2% BSA, 0.01% NaN_3_ and stained with various combinations of fluorochrome-conjugated anti-human mAbs directed to CD45-FITC, CD34-PerCP-Cy5, CD90 (THY)-PE, and PDPN-APC-Cy7 (Biolegend, San Diego, CA, USA) for 30 min at 4°C, after pre-incubation with rabbit IgG (obtained from Sigma, Lenno, Italy) to block non-specific sites. Fluorescence was quantitated on a FACSCanto flow cytometer (BD Biosciences, Franklin Lakes, NJ, USA) equipped with the Kaluza 2.1 software (Beckman Coulter Life Sciences). Cells were gated according to their light-scatter properties to exclude cell debris.

### RNA isolation and quantitative RT-PCR

Total RNA was purified from SF-FLSs and sFBs using Trizol (Life Technologies, Monza, Italy), quantified by NanoDrop spectrophotometry (NanoDrop Technologies, Wilmington, NC, USA), and reverse-transcribed into cDNA on a GeneAmp PCR System 2700 thermal cycler (Applied Biosystems, Milano, Italy) using the SuperScript III Double-Stranded cDNA synthesis kit (ThermoFisher, Waltham, USA). Quantitative RT-PCR (qRT-PCR) was performed on a 7500 Real Time PCR System (Applied Biosystems) in triplicate for each target transcript (Table 2) using the SYBRGreen PCR Master Mix (ThermoFisher, Waltham, USA) and sense/antisense oligonucleotide primers synthesized by Roche (Monza, Italy). Expression data were normalized on the values obtained in parallel for two reference genes (actin-related protein 2/3 complex 1B, *ARPC1B*; Tyrosine 3-Monooxygenase/Tryptophan 5-Monooxygenase Activation Protein Zeta, *YWHAZ*). Gene expression was calculated using the 2-^ΔCT^ method. The sequences of all primer pairs are listed in Table 2.

**Table 2.** Sequences of primers used for RT-PCR

		Forward	Reverse
Housekeeping	*ARPC1B*	AACGAGAACAAGTTTGCTGTG	GATGGGCTTCTTGATGTGC
	*YWHAZ*	TCATGCGGCCTTTTTCCA	TCTGTCTTGTCACCAACCATTCTT
Target genes	*IL6*	AGTGAGGAACAAGCCAGAGC	GGGTCAGGGGTGGTTATTG
	*IL-1β*	TGATGTCTGGTCCATATGAACTG	TGTACAAAGGACATGGAGAACAC
	*TNF-α*	TGGCCCAGGCAGTCAGA	GGTTTGCTACAACATGGGCTACA
	*IL8*	GTGTGAAGGTGCAGTTTTGC	TCTGCACCCAGTTTTCCTTG
	*CCL5*	ACAGCCTCTCCCACAGGTAC	CAATGTAGGCAAAGCAGCAG
	*MCP-1*	TGGAATCCTGAACCCACTTC	AGCAGCAAGTGTCCCAAAGA
	*CXCL5*	TGTTTGCCGCTTAAGCTTTC	TGGCTCACACTATAGTCAATTGC
	*MMP-1*	CTGGGAGCAAACACATCTGA	TTGGAAGGCTTTCTCAATGG
	*MMP-3*	TGGCTCCATGGAATTTCTCT	CGATGCAGCCATTTCTGATA
	*MMP-9*	CAGTCCACCCTTGTGCTCTT	ATCTCTGCCACCCGAGTGTA

### Chondrocytic differentiation

For chondrocyte differentiation assay, SF-FLSs were centrifuged at 300 *g* × 10 min and seeded at 0.5 × 10^6^ cells in 15 ml tubes. Cells were cultured for 28 days (37°C, 20% O_2,_ 5% CO_2_) in a differentiation medium composed of TGF-β1 (10 ng/ml, Peprotech, Waltham, USA) in DMEM-HG (4.5 g/l, Euroclone, Milan, Italy) supplemented with L-ascorbic acid 2-phosphate (50 μg/ml), sodium pyruvate, proline (40 μg/ml, Sigma, Waltham, USA), dexamethasone (10^−7^ M, Sigma), 1% penicillin–streptomycin (Gibco, Waltham, USA), and ITS+premix (Beckton Dickinson, New Jersey, USA), as described [19]. FLSs cultured in DMEM 10% FBS, 1% Pen/Strep, and 1% Glutammine were used as controls. At the end of the culture period, the pellets were washed with PBS and fixed with 4% paraformaldehyde (PAF) for 1 h at RT. After fixation, the pellets were washed three times with H_2_O and then stained with Alcian Blue (AB) (Merck-Millipore, Milano, Italy) for 45 min at RT. After staining, the pellets were washed three times using a solution of ethanol (60% v/v) and acetic acid (40% v/v).

### Statistical analysis

Statistical analyses were performed using the GraphPad Prism 8 software (GraphPad Software, La Jolla, USA). Data are reported as the mean ± s.d. of at least three independent experiments, unless differently specified. Statistical significance was evaluated by performing a two-tailed unpaired Mann–Whitney test. *P* values of <0.05(*), <0.01 (**) and <0.001(***) were considered statistically significant.

## Results

### SF-FLSs in JIA patients with active disease display a sublining-like phenotype

**Figure 1.**
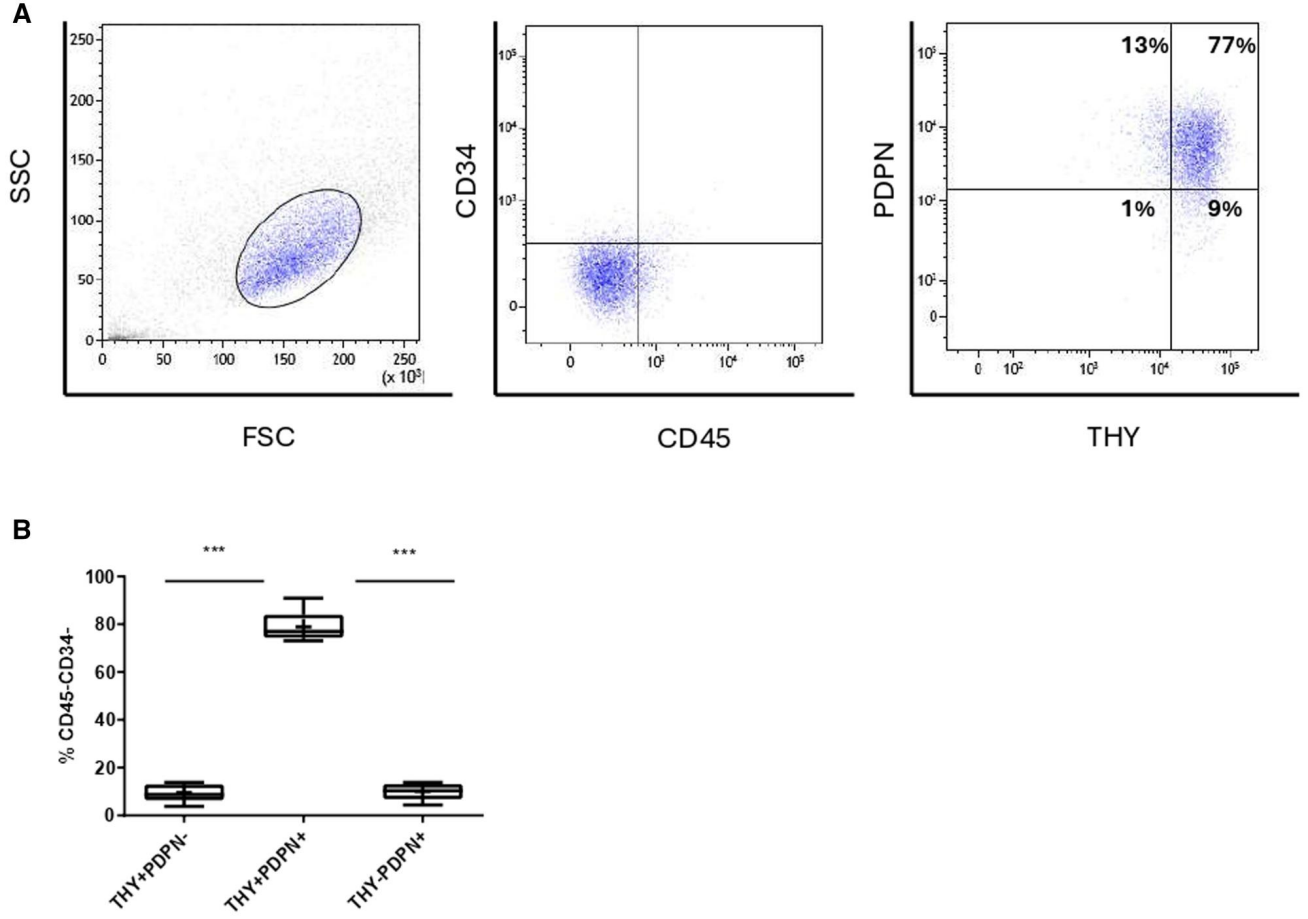
Phenotypic characterization of fibroblast-like synoviocytes isolated from the SF (SF-FLSs) isolated from JIA patients. SF-FLSs were isolated from the SF of seven JIA patients, stained with Abs against CD45, CD34, THY, and PDPN markers, and analysed by flow cytometry. Cells were gated according to their physical properties. (A) Flow cytometry plots of a representative patient. CD45^−^CD34^−^ cells were gated and their positivity for THY and PDPN was evaluated. The number within each plot indicates the percentage of THY^+^ and PDPN^+^ cells in CD45^−^CD34^−^gated cells. (B) Comparative analysis of FLS phenotypic subpopulations. The percentages of CD45^−^CD34^−^THY^+^PDPN^−^, CD45^−^CD34^−^THY^+^PDPN^+^, and CD45^−^CD34^−^THY^−^PDPN^+^ SF-FLSs in all analysed patients were quantified and expressed as a box plot. The boxes comprise the values falling between the 25th and 75th percentiles, horizontal lines represent the median values, and whiskers (lines that extend from the boxes) represent the highest and lowest values for each group, ‘+’ represents the mean values. *P*-value of THY^+^PDPN^+^ relative to THY^+^PDPN^−^ and THY^−^PDPN^+^ ***P < 0.001

To characterize the phenotype of FLSs in the SF of children affected by JIA, total cells were collected from the knee SF aspirates of patients with active disease undergoing arthrocentesis and then cultured to 80% confluence. A homogeneous culture of spindle-shaped cells was obtained after passage 3, confirming previous reports [21]. After the third passage, cells were trypsinized, stained with fluorochrome-conjugated antibodies, and analysed by flow cytometry. Immune cells in the culture were excluded by gating on CD45^−^ cells. The expression of LL- and SL-FLS markers, CD34, CD90 (THY), and PDPN was then assessed. As shown in Fig. 1, the majority of SF-FLSs were negative for both CD45 and CD34 markers. Among CD45^−^CD34^−^ cells, the percentage of THY^+^PDPN^+^ SF-FLSs was significantly higher (mean 79%; range 73%–91%) than that of both THY^+^PDPN^−^ (mean 9.7%; range 3.8%–14%) and THY^−^PDPN^+^ (mean 10%; range 4.4%–14%).

These results indicate that SF-FLSs from JIA patients with active joint disease are CD45^−^CD34^−^THY^+^PDPN^+^, consistent with the phenotype reported for the SL-FLS population in the SM (12; 13) and for SF-FLSs from RA patients [16].

### Gene expression profile of pro-inflammatory cytokines in SF-FLSs

**Figure 2.**
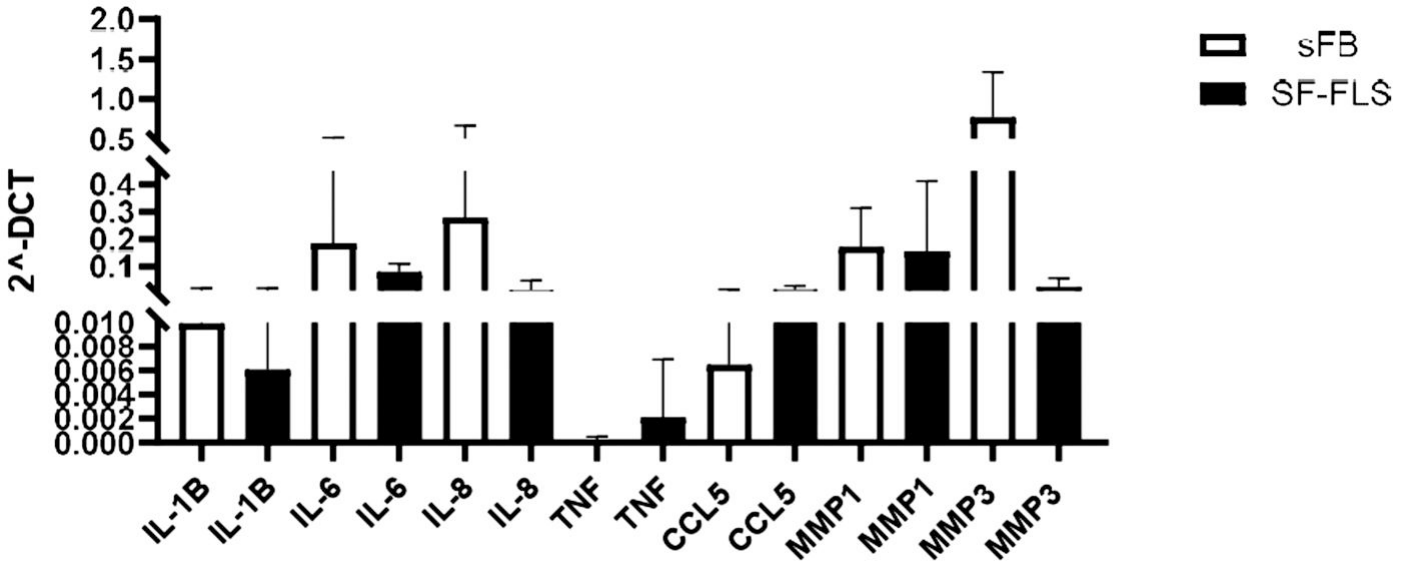
Gene expression of proinflammatory cytokines and MMPs in fibroblast-like synoviocytes isolated from the SF (SF-FLSs) from JIA patients. Transcript expression levels for several proinflammatory cytokines and MMPs were quantified by quantitative RT-PCR (qRT-PCR) and compared between SF-FLSs from JIA patients and sFBs from control group children. Bars represent the mean normalized expression, calculated on the basis of triplicate measurements for each experiment, relative to the values obtained in parallel for two reference genes. Data are presented as 2-ΔCT. sFB: skin fibroblast

To assess whether SF-FLSs are characterized by a pro-inflammatory gene signature typical of SL-FLSs of RA SM, gene expression in cultured SF-FLSs was then analysed by qRT-PCR. We focused on both genes coding for proinflammatory cytokines and chemokines (IL1-β, IL-6, IL-8, TNF-α, and CCL5) and for MMPs (MMP-1, MMP-3) involved in JIA pathogenesis [24; 25]. Transcript levels were normalized on the expression of genes measured in sFBs from five control (CTR) children. Expression of TNF-α and CCL5 was increased in SF-FLSs, although observed differences did not reach statistical significance. In contrast, similar or lower constitutive expression of genes coding for the other tested cytokines and MMPs were detectable in SF-FLSs with respect to sFBs (Fig. 2).

**Figure 3.**
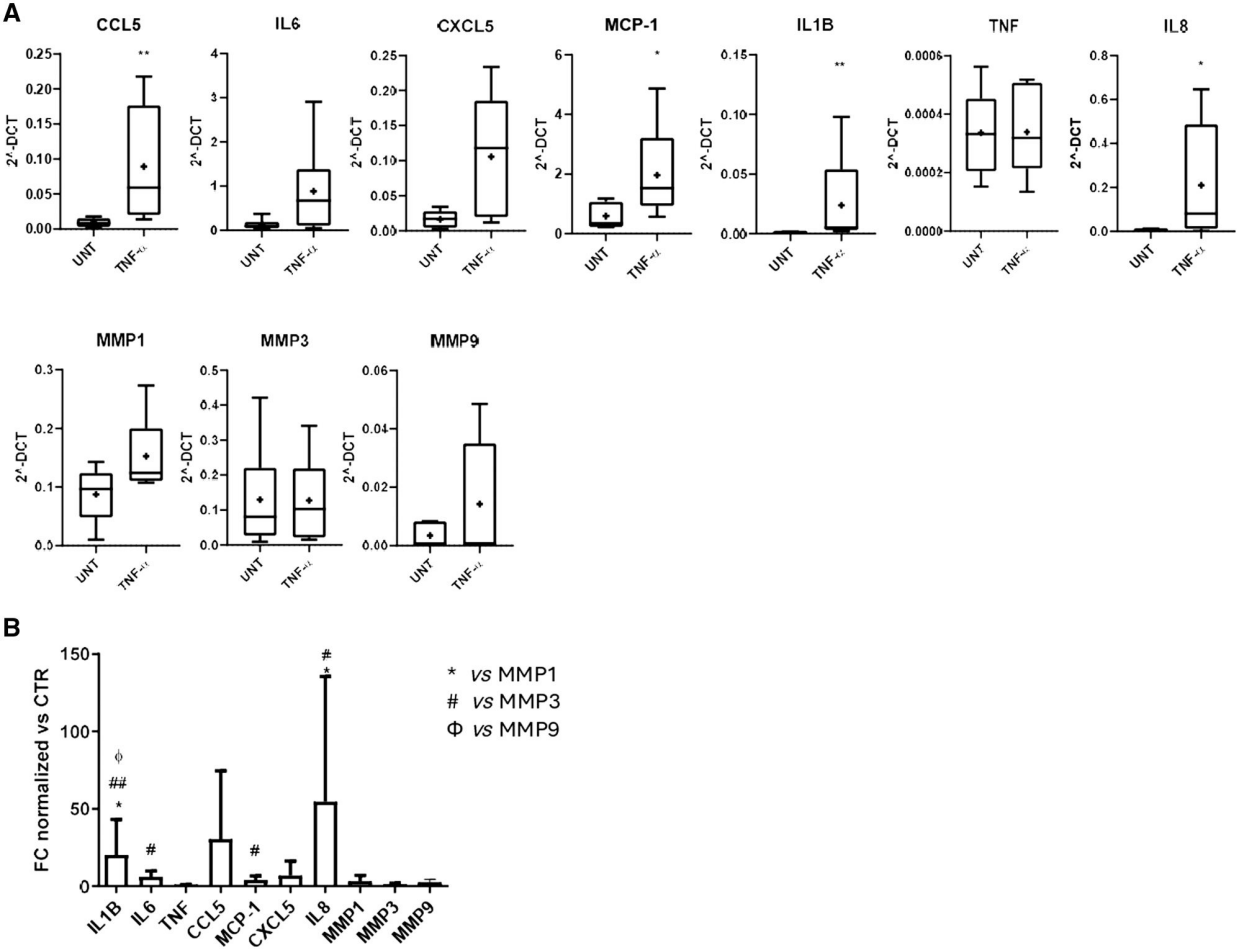
Proinflammatory cytokine and MMP gene expression in fibroblast-like synoviocytes isolated from the SF (SF-FLSs) from JIA patients in response to TNF-α stimulation. SF-FLSs were treated with 10 ng/ml TNF-α for 24 h, and mRNA levels for proinflammatory cytokines and MMPs were quantified by quantitative RT-PCR (qRT-PCR) in total RNA. (**A**) Gene expression in stimulated and untreated (UNT) cells. Data are presented as a box plot, as described in Fig. 1B. *P* values between TNF-α–treated and untreated cells: * *P <* 0.05, ** *P <* 0.01. (**B**) Fold change modulation of cytokine and MMP gene expression. Bars represent the mean normalized expression, calculated on the basis of triplicate measurements for each experiment, relative to the values obtained in parallel for two reference genes (FC = Fold Change normalized on gene expression levels in sFB).*,#, φ *P <* 0.05, ## *P <* 0.01. CTR: control group; FC: fold change; sFB: skin fibroblast

To assess the response of SF-FLSs to a pro-inflammatory stimulus present in the active joints of JIA patients, SF-FLSs were cultured in the presence of 10 ng/ml TNF-α for 24 h, and expression of the mRNA for several cytokines/chemokines and MMPs was then investigated by qRT-PCR. In addition to TNF-α, IL-8, CCL-5, IL-6, IL1-β, MMP-1 and MMP-3, we also measured *MCP-1*, *CXCL5*, and *MMP-9* mRNA levels (Fig. 3). A significant increase in the expression of *IL-8* (*P <* 0.05), *CCL-5* (*P <* 0.01), *IL-1β* (*P <* 0.01), and MCP-1 (*P <* 0.01) coding genes was observed in FLSs stimulated with TNF-α, as compared with unstimulated cells. Higher, although not statistically significantly, expression levels of the *IL-6* and *CXCL-5* genes were also detectable (Fig. 3A). TNF-α treatment induced only a slight and not significant increase in *MMP-1* and *MMP-9* levels, while it did not affect the expression of *MMP-3* (Fig. 3A). In TNF-α–treated cells, the gene expression levels of some cytokines were significantly higher than those of MMPs. Specifically, *IL-1β* gene expression was significantly higher than that of all MMPs, whereas the expression levels of the *IL-6* and *CCL-5* genes were significantly higher with respect to those of *MMP-3*. Finally, *IL-8* mRNA levels were significantly higher than those of *MMP-1* and *MMP-3* (Fig. 3B).

Our data suggest that SF-FLSs can display high proinflammatory activity and low capacity of tissue degradation in the joint environment.

### SF-FLSs can differentiate into chondrocytes *in vitro*

**Figure 4.**
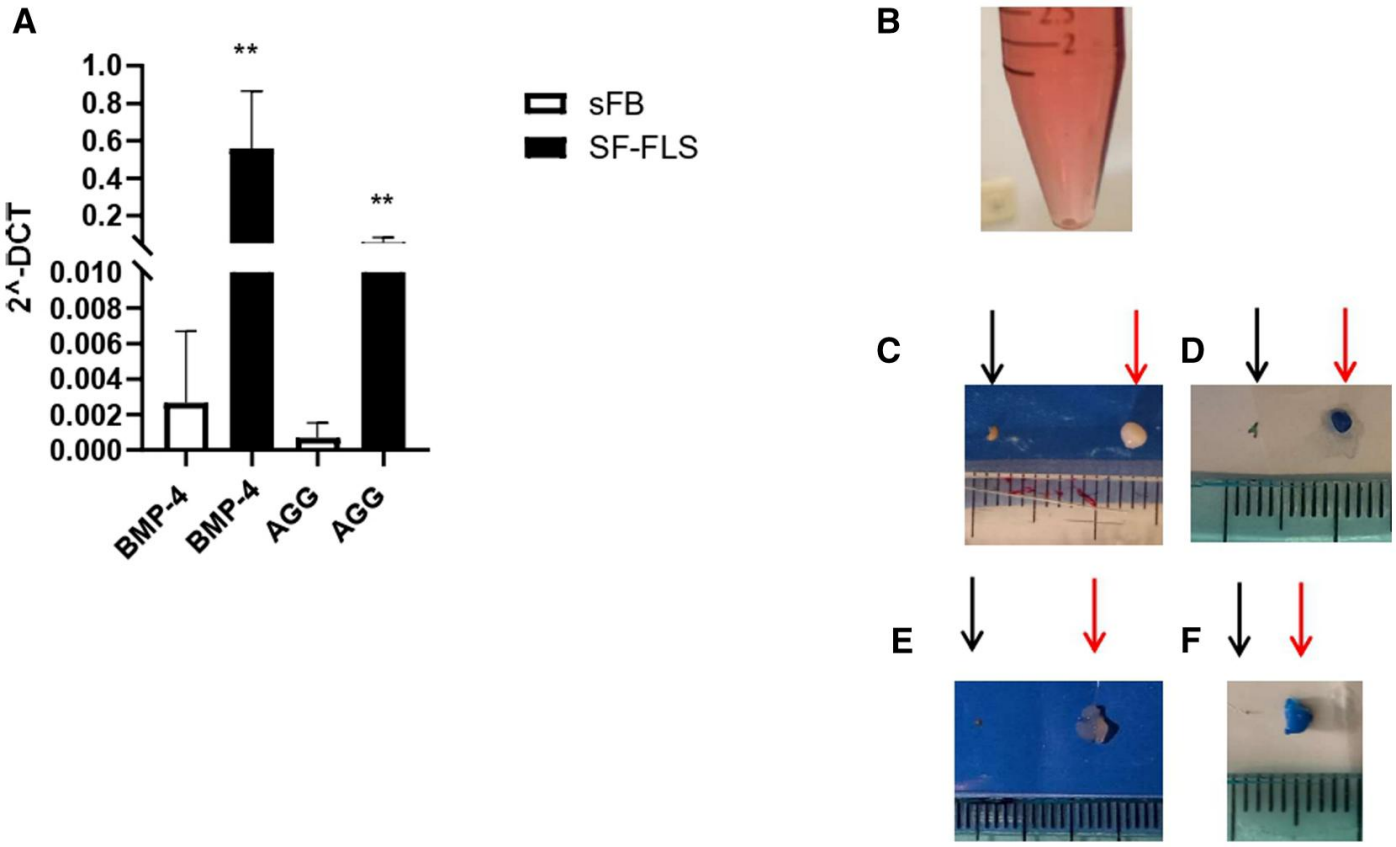
Chondrogenic differentiation potential of fibroblast-like synoviocytes isolated from the SF (SF-FLSs). (A) Expression levels of BMP-4 and AGG were quantified by quantitative RT-PCR (qRT-PCR) in total RNA and compared between SF-FLSs and sFBs. Results are presented as in Fig. 2. *P* values between JIA (SF-FLSs) and control group (sFB) samples:**P < 0.01. (B) Formation of the cell pellet during the 28-day incubation period with the differentiation medium containing TGFβ. (C–F) Representative pictures of chondrocyte differentiation. At the end of the 28-day culture period, the dimension of cell pellets cultured in differentiation and control medium was compared (C, E). Alcian blue staining (D, F) was performed after fixation. (Control pellets, left arrow; differentiated pellet, right arrow). sFB: skin fibroblast

To assess the chondrocyte-differentiation potential of SF-FLSs, the expression of the *BMP-4* and Aggrecan (*AGG*) genes was evaluated (Fig. 4A). SF-FLSs from all patients displayed significantly higher expression of *BMP-4* (*P <* 0.01) and *AGG* (*P <* 0.01) (compared with control SFbs) (Fig. 4A), suggesting a chondrocyte-like phenotype for SF-FLSs, in line with previous data [21].

SF-FLS differentiation into chondrocytes was then evaluated by AB staining to detect proteoglycan production, a characteristic of chondrocytes [19, 26].

SF-FLS cells were centrifuged and cultured as a pellet (Fig. 4B) for 28 days in differentiation medium containing TGF-β (see methods section for details). Cells cultured in complete medium without TGF-β were used as a control. At the end of the culture period, pellets exposed to the differentiation medium appeared larger than those cultured in control medium (Fig. 4C, E), and differentiated cells looked tightly linked to each other as compared with control cells, which were loosely connected. Moreover, SF-FLSs cultured with the differentiation medium exhibited an intense blue or dark-blue colour upon AB staining, confirming chondrogenic differentiation (Fig. 4D, F).

## Discussion

This is the first study showing that SF-FLSs isolated from the joint of JIA patients with active arthritis exhibit a CD45^−^CD34^−^THY^+^PDPN^+^ phenotype and present higher expression of genes coding for several pro-inflammatory cytokines than for MMPs in response to stimuli present in the synovial environment, as well as the capacity to differentiate into chondrocytes.

Double positivity for THY and PDPN was consistently reported as a typical feature of SL-FLSs in the SM of patients with RA [12]. Conversely, data in the literature on CD34 expression are contrasting. Negativity of this marker was in fact reported by several groups as a hallmark of LL-FLSs in the SM of RA patients [13, 14, 27], whereas the existence of a CD34^−^ FLS subpopulation in the SL region of the SM cell subset was reported by Zhang *et al.* [27]. A population of CD34^−^ THY^+^ cells was also observed in the SL-SM of RA patients, forming a perivascular zone around blood vessels, especially near lymphocyte-enriched areas [13]. In addition, our data are consistent with and extend the previous results for JIA reported by Kostar *et al.* [16] in RA patients, showing the presence of CD45^−^CD34^−^THY^+^PDPN^+^ FLS in the SF.

Given the reported proinflammatory and tissue-degrading roles specifically played by SL-FLSs and LL-FLSs isolated from the SM of RA patients and murine arthritis models [9], it was critical to investigate SF-FLS functional properties to elucidate their possible subtype of origin. We demonstrated higher expression of TNF-α and CCL5 pro-inflammatory cytokines in SF-FLSs as compared with sFBs used as control cells, although the increase did not reach statistical significance. The finding that no differences were observed in the constitutive expression of the mRNAs coding for the other tested cytokines, such as IL1-β, IL-6, and IL-8, may probably in part depend on the prolonged culture conditions required for FLS isolation. These results are in contrast with data reported by Brescia and colleagues, who showed an increase of IL-6 and IL-8 in SF-FLSs compared with control FLSs from children undergoing orthopedic procedures [9]. These discrepancies may be accounted for by the different methodologies used—gene expression in our study and protein secretion in Brescia’s report—or by the different types of control fibroblasts. Our choice of using sFBs as a control was due to the difficulty of collecting SF samples from healthy children or from patients undergoing minor orthopedic procedures exclusively for research purpose and not as part of the therapeutic strategy, because of ethical reasons. In addition, previous studies in the literature have reported the use of sFBs for comparison with SM-derived FLSs in RA patients, showing the suitability of this experimental model for the analysis of gene expression and functions [28]. Similarly to what was found in our study, no differences in the mRNA levels of genes coding for IL1-β, IL-6, and IL-8 were detected between SM-FLSs and sFBs in RA [28]. Interestingly, we demonstrated that SF-FLS stimulation with the pro-inflammatory stimulus, TNF-α, which is present in the joint inflammatory environment in JIA patients and involved in disease pathogenesis [29], induced significantly increased expression of IL-8, MCP-1, IL-1β, CCL-5, IL-6, and CXCL5-coding mRNA levels without affecting expression of MMPs. Noteworthily, some of the overexpressed cytokines, such as IL-6, MCP-1, and CCL5, have been reported as typical of the SL-FLSs in the SM of mice models [12]. These data suggest phenotypic and functional similarities between SF-derived FLSs and SL-FLSs described in the RA SM.

Another important result of this study is the demonstration of SF-FLS constitutive expression of high levels of the *AGG* and *BMP-4* genes, known for their role in chondrogenesis, as compared with control sFBs, in line with data by Brescia *et al.* [21]. Autoreactive T cells able to recognize Aggrecan molecules were described in both RA [30] and JIA [31], suggesting its contribution in disease pathogenesis, whereas BMP-4 was shown to cause chondrocyte hypertrophy during endochondral bone formation facilitating the bone overgrowth seen in arthritic joints [32]. Moreover, we demonstrate for the first time that CD45^−^CD34^−^THY^+^PDPN^+^ cells from the SF of JIA patients have the ability to differentiate into chondrocyte-like cells.

It is critical to point out that our study was carried out on a small and heterogeneous cohort of JIA patients, which may represent a potential limitation. The ILAR classification of JIA mostly relies on patient clinical characteristics at presentation (e.g. the number of joints affected), without taking into account genetic, molecular or immune features. However, genetics and/or epigenetics may also differ in the various ILAR JIA subtypes, potentially underlying specific immuno-pathogenic mechanisms, and a new molecular-based classification system has also been proposed [33, 34]. Some inherited genes for disease susceptibility and severity have, in fact, been identified in JIA, among which HLA molecule–coding gene polymorphisms have been shown to confer the strongest genetic effect, showing both differences and similarities across subtypes. For instance, the HLA-DRB1*01 haplotype is positively associated with oligoarticular JIA, whereas oligoarticular and poliarticular JIA share a strong association with the HLA-DRB1*08 haplotype, and enthesitis-related JIA is associated with the HLAB27 haplotype [4, 35–38]. Although genetic and molecular differences between the oligoarticular and polyarticular subtypes may be limited, systemic JIA patients present substantial diversity, exhibiting not only clinical systemic inflammatory features similar to those of autoinflammatory diseases, but also a unique transcriptional profile and genetic architecture, not sharing heritable risk factors with oligo or polyarthritis, even within the *HLA* region (e.g. it shows association with the HLA-DRB1*11 haplotype), as determined by transcriptomic and large-scale genomic studies [39, 40]. However, the development of chronic arthritis, a feature common to all JIA subtypes and one of the main criteria for JIA definition, also occurs in more than half of patients with systemic JIA [40]. Based on these considerations, it was reasonable to expect that FLS phenotype and functionality could be differentially affected in patients classified into the three different ILAR subtypes (oligoarticular, polyarticular and systemic). Hence, the finding that FLSs isolated from all SF samples exhibit phenotypic and gene transcription similarities is of particular relevance, indicating that SF-FLS–dependent pathogenic mechanisms involved in joint flare occurrence might be shared among distinct JIA subgroups, independently of their clinical, genetic, and epigenetic characteristics. The possibility that the FLS response in systemic JIA (sJIA) may not be differentially affected with respect to the other subtypes is further supported by the demonstration that specific genetic variations have been mainly identified in immune-regulating genes [40].

Another potential limitation of the study is represented by the systemic medications to which most enrolled patients were exposed at the time of sampling, which could have influenced the proinflammatory and/or tissue destructive signatures of the FLSs, representing a potential confounder. Therapeutic response may be vary widely among the various JIA subtypes, and even within patients of the same subtype [40]. Divergent results have been reported in the literature on the effects of DMARDs and biologic agents on FLSs from RA patients. DMARDS, such as MTX, have been shown to decrease MMP and proinflammatory cytokine production by FLSs [41], but also to increase the gene expression of some proinflammatory cytokines (e.g: CCL20 but not IL-6) and decrease RNA levels of MMP3 in FLSs as well as the PDPN marker, compared with controls [42]. In addition, some biologic agents, such asanti-CD20 and CTLA4-Ig, exclusively target immune cells and do not interfere with fibroblast functions, while anti-cytokine treatments, such as TNF inhibitors and IL-6 receptor blockers, interfere with the fibroblast contribution to RA, as they are activated by both cytokines [43]. Due to the small sample size of our patient cohort, it was not possible to fully assess the potential confounding effects of systemic medications on the FLS proinflammatory and/or tissue-destructive signature in this study. However, it is important to note that all tested patients were enrolled during active arthritis flares, suggesting comparable pathological characteristics of the joints at the time of sampling. In addition, given the occurrence of disease relapse, it is conceivable that. the therapeutic agents may no longer have been exerting their anti-inflammatory effects at the time of patient enrolment. Several factors could counteract the effects of systemic therapy. For example, other stimuli present in the local synovial microenvironment and effective on FLSs, such as the extracellular matrix or interactions with other cell types, could sustain FLS activation despite treatment. Alternatively, FLSs may develop resistance or reduced responsiveness to the drugs due to the chronicity of inflammation. Furthermore, systemic therapies do not always fully restore the normal function of synovial cells [44, 45]. Finally, it is also possible that the prolonged culture conditions in drug-free medium required for FLS isolation, may have diluted the effects of treatment. However, drugs effects on SF-FLS phenotype can not be excluded. In conclusion, this exploratory study provides an important characterization of the phenotypic and functional properties of FLSs in the SF from the active joints of JIA patients, suggesting that these cells can display proinflammatory activity and low capacity for tissue degradation similar to SL-FLSs from the SM of adult arthritis patients and murine models of RA, thus improving our understanding of the mechanisms underlying JIA pathogenesis. In addition, our results highlight the potential of SF-derived FLSs as an *in vitro* model for studying synovial and articular cartilage tissues, offering an opportunity for more detailed characterization of these cells. A follow-up validation study will be carried out on an independent larger cohort of patients to confirm these data and determine whether any difference in FLS phenotype and functionality, undetected in the present report due to the small sample size of the patient cohort analysed, exist among the various JIA subtypes and in response to different therapeutics. The inclusion in the follow-up study of treatment-naïve patients will also help to exclude the potential confounding effects of medications.

The protocol of the study was reviewed and approved by the Ethics Committee of the Region Liguria (Approval 165/2018) and by the Ethics Committee of the Gaslini Institute for biobanc ‘Istituzione di biobanca per pazienti affetti da malattie reumatiche dell’età pediatrica’ (Approval 06/05/2004). The procedures were carried out according to the approved guidelines and in adherence to the general ethical principles set forth in the Declaration of Helsinki. Written informed consent to participate in the study was obtained from the parents or the patient’s legal guardian prior to sample collection.

## Supplementary Material

keaf266_Supplementary_Data

## Data Availability

The data underlying this article will be shared on reasonable request to the corresponding author.
